# Action of Actomyosin Contraction With *Shh* Modulation Drive Epithelial Folding in the Circumvallate Papilla

**DOI:** 10.3389/fphys.2020.00936

**Published:** 2020-07-31

**Authors:** Sushan Zhang, Jong-Min Lee, Adpaikar Anish Ashok, Han-Sung Jung

**Affiliations:** Division in Anatomy and Developmental Biology, Department of Oral Biology, Brain Korea 21 PLUS Project, Taste Research Center, College of Dentistry, Yonsei University, Seoul, South Korea

**Keywords:** circumvallate papilla, sonic hedgehog, epithelial folding, actomyosin, focal adhesion kinase

## Abstract

The mouse tongue possesses three types of gustatory papillae: large circumvallate papillae (CVP), foliate papillae (FOP) and fungiform papillae (FFP). Although CVP is the largest papilla and contain a high density of taste buds, little is known about CVP development. Their transition from placode to dome-shape is particularly ambiguous. Understanding this phase is crucial since dome-shaped morphology is essential for proper localization of the imminent nerve fibers and taste buds. Here, we report actomyosin-dependent apical and basal constriction of epithelial cells during dynamic epithelial folding. Furthermore, actomyosin-dependent basal constriction requires focal adhesion kinase to guide dome-shape formation. Sonic hedgehog (Shh) is closely associated with the differentiation or survival of the neurons in CVP ganglion and cytoskeletal alteration in trench epithelial cells which regulate CVP morphogenesis. Our results demonstrate the CVP morphogenesis mechanism from placode to dome-shape by actomyosin-dependent cell shape change and suggest roles that Shh may play in trench and stromal core formation during CVP development.

## Introduction

Circumvallate papillae (CVP) are located on the dorsal surface of the posterior tongue in mammals (Jitpukdeebodintra et al., 2003; Chandrashekar et al., 2006; Barlow and Klein, 2015). Despite the importance of CVP as the largest gustatory papilla and that possess a high density of taste buds, our understanding of its cellular development is imperfect. Recently, molecular regulation of FFP development have substantially been revealed. Previous studies showed that Shh, Bmps, and Wnts regulate FFP patterning and morphogenesis in early stages. However, the functions that these genes play in CVP development have not been fully investigated (Hall et al., 2003; Mistretta et al., 2003; Liu et al., 2004, 2007; Zhou et al., 2006; Iwatsuki et al., 2007; Beites et al., 2009).

Previously, various genes associated with CVP defects have been reported. Mesenchymal Fgf10 is essential for the maintenance of Lgr5/Sox2-positive CVP epithelial progenitor cells, which is consistent with the absence of CVP structures in Fgf10-/- mice (Petersen et al., 2011; Zhang et al., 2018). In *Pax9*^–/–^ mice, trench formation in CVP and FOP is retarded (Kist et al., 2014). Morphological abnormalities of CVP have been observed in *Six1*^–/–^*Six4*^–/–^, *Ripply3*^–/–^ and *Tbx1*^–/–^ mice correlated with glossopharyngeal nerve innervation failure. These abnormalities correlated with glossopharyngeal nerve innervation failure suggest a potential relationship between innervation and morphogenesis (Guth, 1957; Suzuki et al., 2011; Okubo and Takada, 2015). Although correlations were recorded, no single gene has been identified as the direct regulator of the morphological change from placode to dome-shape. Nor did the prior investigations into this transition yield a working hypothesis for the responsible mechanism (Petersen et al., 2011).

The placode to dome-shape transition is based on epithelial folding, including invagination and evagination (Pearl et al., 2017). Invagination has been studied in various organs such as teeth and salivary glands (Jaskoll et al., 2004; Li et al., 2016; Panousopoulou and Green, 2016). However, the invagination mechanisms, including apical constriction and basal relaxation, are strictly applicable to the monolayer epithelial folding seen in neural tubes and lenses (Sai and Ladher, 2008; Sawyer et al., 2010; Martin and Goldstein, 2014; Pearl et al., 2017). Conversely, the CVP placode is composed of multiple layers of epithelial cells at E12.5. The transition from dental placode to tooth bud is an example of a multilayered epithelial folding. It has been explained using the suprabasal intercalation model, which is applicable to the development of other ectoderm-derived organs. Invagination in the suprabasal intercalation model is led by parallel suprabasal cells intercalation with region-specific E-cadherin (Panousopoulou and Green, 2016; Pearl et al., 2017). However, according to E-cadherin expressing cells and their cell orientation, both parallel suprabasal cells and region-specific E-cadherin were absent in the CVP placode. Because monolayered and multilayered epithelial folding models could not explain invagination in CVP, we analyzed each cell shape during early CVP development that potentially lead to morphological changes by epithelial folding (Pearl et al., 2017). Most of the previously reported epithelial folding was influenced by cell shape changes, especially actomyosin-dependent apical/basal constriction (Pearl et al., 2017). In this study, we confirmed that both apical and basal constriction during epithelial folding in developing CVP were actomyosin-dependent. Invagination is necessary for various organogenesis phenomena, however, genes responsible for regulating invagination have not been reported for CVP. Previous studies have reported that Shh regulates invagination during the transition from dental placode to tooth bud through changes in the epithelial cell shape. Similar to what is exhibited in dental epithelium, Shh is continuously expressed in CVP epithelium from E12.5 through adulthood (Mistretta et al., 2003; Iwatsuki et al., 2007; Kim et al., 2009). Reduced Shh expression, which led to impaired trench formation in *Pax9*^–/–^ mice, indicates a potential correlation between epithelial Shh and CVP morphogenesis (Kist et al., 2014). However, morphological defects in CVP has not been reported in Shh pathway-inhibited embryonic tongue cultures (Hall et al., 1999, 2003; Mistretta et al., 2003; Liu et al., 2004; Iwatsuki et al., 2007).

In this study, basal constriction-dependent evagination was observed in the dome-shaped region of the developing CVP. Focal adhesion kinase (FAK) was necessary for basal constriction in outward epithelial folding (evagination) and for proper adherence to the underlying extracellular matrix. This mechanism is highly conserved and established in both the midbrain-hindbrain boundary and the developing tooth germ (Gutzman et al., 2008, 2018; Yamada et al., 2019).

The CVP placode to dome-shape transition happens simultaneously with innervation (Jitpukdeebodintra et al., 2003; Petersen et al., 2011; Kist et al., 2014). Nerve fibers of gustatory nerve enter the developing tongue mesenchyme at E12.0 and reach the tongue epithelium at E13.5 As the gustatory nerve innervates CVP, glial cells in the glossopharyngeal nerve are derived from neural crest cells (NCCs) and neurons in the glossopharyngeal nerve are derived from epibranchial placode-originated neuroblasts. Previous studies indicated that the underlying mesenchyme of CVP is NCCs-derived (Liu et al., 2012). Moreover, nerve fibers in the underlying mesenchyme of CVP were stained by PGP9.5 according to our results. The population of neuroblasts was further confirmed by expression of Neurogenin-2, an essential transcription factor for neuronal development in the glossopharyngeal nerve (Fode et al., 1998; Harlow and Barlow, 2007; Okubo and Takada, 2015; Fan et al., 2019).

The underlying mechanisms of dome-shape and trench formation in developing CVP were investigated in this study. The epithelial folding responsible for the dome and trench formations was actomyosin-dependent. Moreover, basal constriction requiring FAK led to evagination in the dome-shape region. Disruption of trench formation and stromal core morphology was observed after inhibition of Shh pathway by Cyclopamine. Invagination in the trench region was regulated by Shh through modulating apical constriction. Moreover, Shh was found impacted in stromal core formation by regulating differentiation or survival of neurons in CVP ganglion.

## Materials and Methods

All experiments were performed according to the guidelines of the Intramural Animal Use and Care Committee of the College of Dentistry, Yonsei University.

### Animals

Adult ICR mice were housed in a temperature-controlled room (22°C) under artificial illumination and 55% relative humidity with access to food and water *ad libitum*. Embryos were obtained from time-mated pregnant mice. E0 was the day when the presence of a vaginal plug was confirmed. Embryos at each developmental stage (E12.5, E13.0, E13.5) were used in this study.

### *In situ* Hybridization

*In situ* hybridization of tongues was performed as previously described (Kim et al., 2009). Embryonic tongues were dissected and fixed in 4% paraformaldehyde (PFA), dehydrated in methanol at -20°C. After rehydration, tissue went through proteinase K treatment and subsequent 0.25%glutaraldehyde in 4%PFA, then prehybridized in hybridization solution at 68°C for 2 h and hybridized with Digoxigenin-labeled RNA probes for overnight at 68–70°C. Mouse complementary *shh/ptch1*-inserted plasmids were used for synthesizing probes.

### *In vitro* Organ Culture

The tongue was isolated from E12.5 mouse embryos and cultured on a 1.0 μm Nucleapore Track-Etch Membrane (Whatman, Pittsburg, PA, United States) in the medium at 37°C and 5%CO_2_ for 48 h using culture method reported by Trowell. The culture medium (DMEM, Invitrogen, United States) was supplemented with 10% fetal bovine serum (FBS) (Invitrogen, United States) and 1% penicillin/streptomycin and was renewed every 24 h.

### Histology and Immunofluorescence

Samples were fixed in 4% paraformaldehyde and processed until paraffin using standard procedures. Sections (4 μm) were prepared for hematoxylin/eosin staining and immunostaining. After citrate buffer (pH 6.0), Specimens were blocked using 1% goat serum or 5% bovine serum albumin in PBS, incubated with antibodies against endothelin receptor B (1:50, Abcam, United Kingdom), PGP9.5 (1:100, Abcam, United Kingdom), fibronectin (1:100, BD Bioscience, United States), E-cadherin (1:100, R&D Systems, United States), phosphomyosin light chain II (1:50, Cell Signaling Technology, United States), β-catenin (1:100, Santa Cruz, United States), and ZO-1 (1:100, Invitrogen, United States). Sequentially incubate with a secondary antibody (1:200, Invitrogen, United States) and stained with DAPI to visualize nuclei. All specimens were observed by confocal microscopy (YOKOGAWA CQ1, Japan). At least 10 mice were examined in each experiment.

### Inhibitor Treatment

The medium was supplemented with 3μg/ml Blebbistatin, myosin II phosphorylation inhibitor (Sigma Aldrich, United States); 0.75 μM PF-573228, FAK inhibitor (Cayman Chemicals, United States), and 20 μM Cyclopamine, Smoothened inhibitor (Toronto Research Chemicals, Canada). The above chemicals were dissolved in Dimethyl Sulfoxide (DMSO; Sigma Aldrich, United States) for stock until use. Vehicle (DMSO)-treated tongues from the same littermates were used as controls. To minimize effects other than cytoskeleton, drug treatments were performed for the first 24 h.

### Tissue Recombination

Tongues were dissected from E13.5 mouse embryos and kept in ice-cold PBS. Tissue was cut into approximately 200 μm-thick slices use microtome blades. After 60-min incubation in 2.2 U/ml Dispase II (neutral protease, grade II) at 37°C, the epithelium and mesenchyme of CVP were manually separated by forceps and washed in basal medium containing 10% FBS. After 1 h/3 h of standard culture, the epithelium was transferred to PBS and photographed. At least 12 embryos were examined for this experiment.

### Recombination Experiment and Bead Implantation

E12.5 embryonic tongues were dissected and incubated in 2.2 U/ml Dispase II for 50 min at 37°C and washed in basal medium containing 10% FBS. Tongue epithelium and mesenchyme were gently separated in cold medium. The epithelium was placed 180° rotated anterior-posterior. Heparin beads (100–200 mesh; Sigma Aldrich, United States) soaked with 100 μg/ml FGF10 protein (6224-FG-02S; R&D Systems, United States) were implanted between the recombined CVP epithelium and non-CVP mesenchyme at E12.5 and cultured for 48 h as in both groups. The CVP epithelium and underlying mesenchyme combined at original as control. Specimens from 4 embryos were examined for each group.

### Cellular Analysis

400X z-stack images of β-catenin-stained epithelial cells in developing CVP were measured and analyzed by Fiji (Schindelin et al., 2012). Category of cells with contact with basal lamina (basal cells), without contact (suprabasal cells). The maximum cross-sectional area of cells was measured to avoid artifacts. Basal width: width of cell attachment to the basal lamina, apical width: perpendicular to the cell long axis at the apical 20% site. Apical/basal ratio = apical width/basal width. Width/length ratio = width/length. The angle between the cell long axis and its projection into the flanking epithelium plane was measured. Parallel (0°–30°, 150°–180°), oblique (30°–60°, 120°–150°) and vertical (60°–120°) were used for categorize angles (Panousopoulou and Green, 2016; Yamada et al., 2019). 120 cells were counted for each analysis from 3 different specimens for each stage (*n* = 120). Significance was assessed by the *t* test. Data were expressed as the mean ± SD.

## Results

### Epithelial Folding Involves Cytoskeletal Alteration

**FIGURE 1:**
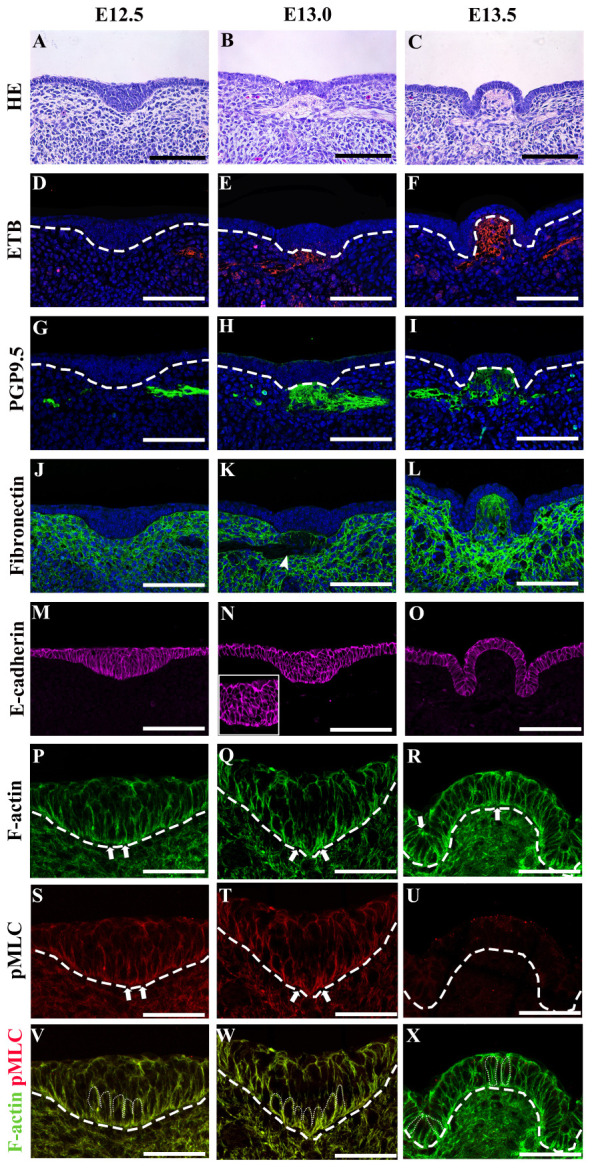
**(A)** The circumvallate papilla (CVP) epithelium at E12.5 is a stratified placode, while non-CVP epithelium is monolayered. **(B)** At E13.0, epithelium started to fold outward at the medial region of the placode. A low cell density region is observed in the underlying mesenchyme at E13.0. **(C)** Dome-shape and lateral trenches are observed with monolayered or pseudostratified CVP epithelium at E13.5. **(D,E)** Endothelin receptor B (ETB) is located in the lateral tongue mesenchyme at E12.5 and underlying mesenchyme of CVP at E13.0. **(F)** ETB is detected in the stromal core of CVP at E13.5. **(G,H)** Protein gene product 9.5 (PGP9.5) is located in the lateral tongue mesenchyme at E12.5 and the underlying mesenchyme of CVP at E13.0. **(I)** The stromal core of CVP is marked by PGP9.5 at E13.5. **(J)** Fibronectin is located throughout the tongue mesenchyme at E12.5. **(K)** Fibronectin density is relatively low in the presence of neuroblasts (marked by ETB) at E13.0 (according to S1C) (arrowhead). **(L)** Fibronectin localization in the stromal core is relatively weaker than the surrounding mesenchyme at E13.5. **(M)** E-cadherin is located throughout the entire epithelium at E12.5. **(N)** E-cadherin is relatively enriched in basal and suprabasal cells of placodes at E13.0. **(O)** At E13.5, slight enrichment of E-cadherin is found in suprabasal cells. **(P)** F-actin accumulation is detected at the basal site of basal cells at E12.5. **(Q)** At E13.0, F-actin strongly accumulates at the basal site of basal cells in the medial region of CVP placode. **(R)** F-actin is enriched at the basal (site in the dome-shape region and at the apical site of the trench region epithelial cells at E13.5. **(S,T)** Phosphomyosin light chain II (pMLC) is enriched at the basal site of basal cells at E12.5 and strictly accumulated at the basal site of the medial region at E13.0. **(U)** pMLC is weakly detected in CVP epithelium at E13.5. **(V–X)** pMLC is co-accumulated with F-actin at E12.5 and E13.0 but not E13.5. Scale bar = 100 μm in panels **(A–O)**, 50 μm in panels **(P–X)**. Arrows indicate the cells which go through apical and basal constriction. Arrowhead indicates the low-density region of fibronectin localization. Thick dotted lines mark the border between developing tongue epithelium and mesenchyme. Thin dotted lines indicate individual cell shapes which go through apical and basal constriction.)

The multilayered CVP placode and monolayered non-CVP epithelium were observed in E12.5 mouse tongue (Figure 1A). At E13.0, CVP epithelium started to fold outward at the medial region of the placode and a low cell density region was observed in the underlying mesenchyme (Figure 1B). Dome-shaped formations and lateral trenches were formed at E13.5 with monolayered or pseudostratified CVP epithelium and stromal core (Figure 1C). The enteric nervous system precursor marker (Baynash et al., 1994; Hosoda et al., 1994), endothelin receptor B (ETB), was located in both the lateral tongue mesenchyme at E12.5 (Figure 1D) and the underlying mesenchyme of CVP placode at E13.0 specifically (Figure 1E). ETB was intensively localized in the stromal core at E13.5 (Figure 1F). Similarly, neuronal marker PGP9.5 was detected in the tongue mesenchyme distant from CVP at E12.5 (Figure 1G). PGP9.5 was observed underneath the mesenchyme of CVP at E13.0 (Figure 1H). At stage E13.5, PGP9.5 was detected in the stromal core (Figure 1I). Furthermore, ETB and PGP9.5 were co-localized at E13.0 (Supplementary Figure S1A). Moreover, neural cell adhesion molecule (NCAM) was detected in the nerve endings and cell bodies inside the stromal core at E13.5. A previous study reported that NCAM is detected in nerves innervated the adult CVP (Supplementary Figure S1B) (Nosrat et al., 2012). Fibronectin was markedly downregulated within the ETB located region at E13.0 (Figure 1K, arrowhead, Supplementary Figure S1C). At E13.5, fibronectin was more weakly displayed in the stromal core than in the surrounding mesenchyme (Figure 1L). E-cadherin located throughout the tongue epithelium, including CVP at E12.5 (Figure 1M). At E13.0, no significant difference of E-cadherin was observed when compared to E12.5 (Figure 1N). At E13.5, slightly prominent E-cadherin localization was observed in peridermal cells (Figure 1O). F-actin accumulated slightly at the basal site of basal cells at E12.5 (Figure 1P). Specifically, F-actin was strongly accumulated at the basal site of the medial region of the placode at E13.0 (Figure 1Q). Also, F-actin was slightly enriched at the apical site of the trench region as well as the basal portion of dome-shape region at E13.5 (Figure 1R). The F-actin intensity differences between the apical and basal site of epithelial cells were confirmed (Table 1). pMLC was enriched basally in basal cells at E12.5 and E13.0 (Figures 1S,T). pMLC enrichment was not detected in CVP epithelium at E13.5 (Figure 1U). Interestingly, F-actin and pMLC co-accumulated in developing CVP epithelium (Figures 1V–X) at E12.5 and E13.0 but not E13.5, which suggested that active actomyosin contraction occurred during CVP morphogenesis. Proliferative and apoptotic cells were not detected in CVP epithelium; proliferative cells were observed in the non-CVP epithelium and tongue mesenchyme including CVP mesenchyme from E12.5 to E13.5 (Supplementary Figures S1D–I). Neurogenin2, a neuroblast marker, was expressed in the underlying mesenchyme of CVP from E12.5 to E13.0, similar to ETB and PGP9.5 localization (Supplementary Figures S1J,K). These results indicated that the neuronal components supposed to be the neurons of the CVP ganglion and nerve fibers of gustatory neurons (Fode et al., 1998; Okubo and Takada, 2015).

**TABLE 1:** Fluorescent intensity of F-actin between apical and basal site of epithelial cells.

	**Stages groups**	**Apical intensity basal intensity of F-actin**
Not defined	E12.5	0.99 ± 0.093
Medial	El3.0	0.85 ± 0.112
	El3.5	0.99 ± 0.099
	Control	0.89 ± 0.105
	Blebbistatin	1.01 ± 0.104
	PF-573228	0.97 ± 0.114
Lateral	E13.0	1.21 ± 0.120
	E13.5	1.18 ± 0.112
	Control	1.27 ± 0.095
	Cyclopamine	0.97 ± 0.110

*<1 indicates apical site weaker than basal site. ≈1 indicates no significant difference is observed. >1 indicates apical site stronger than basal site.*

### Cell Shape Changes Following Epithelial Folding

**FIGURE 2:**
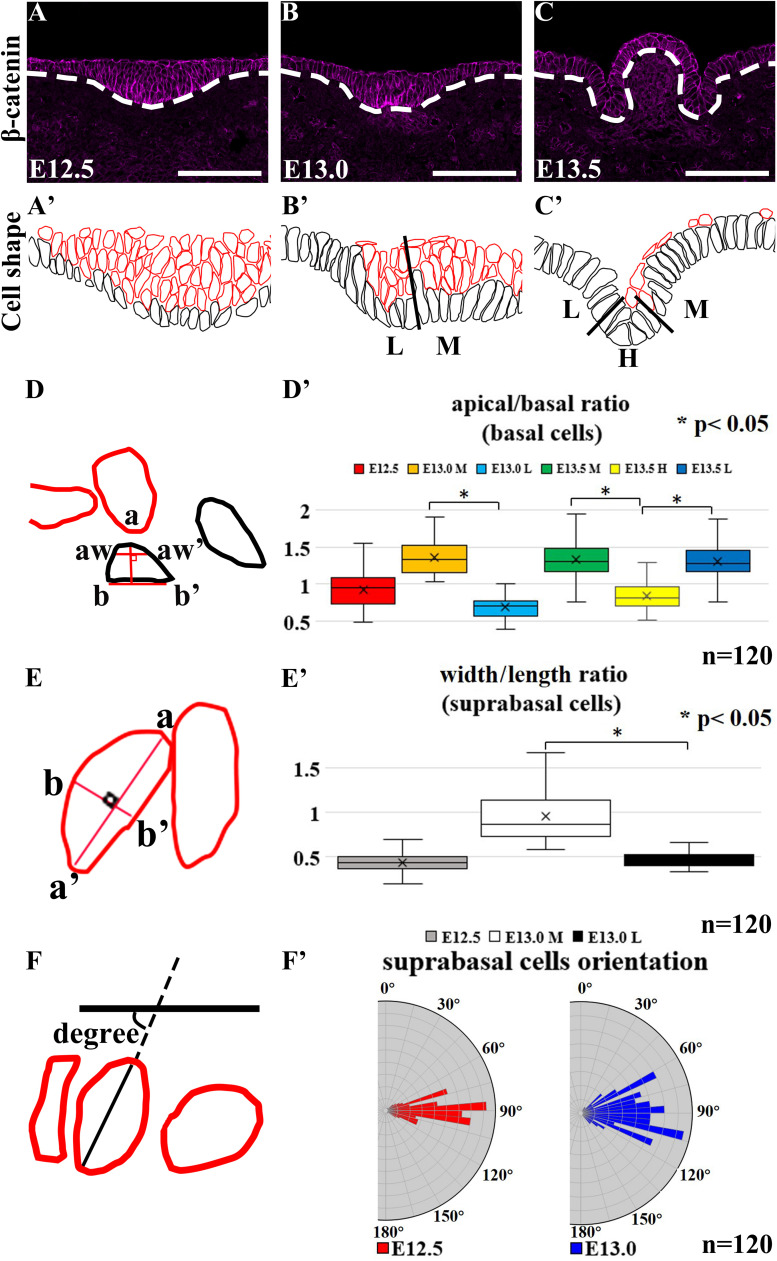
**(A’–C’,D–F)** Black-colored are basal cells while red-colored are suprabasal cells. **(A,A’)** Basal and suprabasal cells in CVP are columnar at E12.5. In medial region “M,” basal cells become constricted at the basal site, while suprabasal cells become oval-shaped at E13.0. **(B,B’)** Epithelial cells in the lateral region “L” of the CVP remain columnar. **(C,C’)** Cells in the medial and lateral regions become columnar with basal constrictions at E13.5. Cells in the hinge region “H” of CVP show apical constriction at E13.5. **(A,A’,D’)** At E12.5, all basal cells were columnar. **(B,B’,D’)** At E13.0, mediobasal cells become basal constricted, while laterobasal cells are relatively columnar. **(C,C’,D’)** At E13.5, basal constriction is found in basal cells in the medial and lateral regions, while hinge region cells show strong apical constriction. **(A,A’,B,B’,E’)** At E12.5, all suprabasal cells are elongated, but suprabasal cells in the medial region become oval-shaped at E13.0; suprabasal cells in the lateral region remain elongated. **(F,F’)** The angles between the suprabasal cell long axis to the plane of flanking epithelium are 80°–100° at E12.5 and 70°–110° at E13.0 in vertical-to-oblique range (45°–135°). Scale bar = 100 μm. White dotted lines mark the border between the developing tongue epithelium and mesenchyme. *n* = 120 cells (from three different littermates for each stage). *P < 0.05.

Basal cells were colored black while suprabasal cells were colored red in cell shape drawing based on β-catenin staining (Meng and Takeichi, 2009) (Figures 2A–C,A’–C’). At E12.5, epithelial cells in CVP were columnar (Figures 2A,A’). The medial region was designated as “M,” the hinge region as “H” and lateral as “L.” At E13.0, basal constriction occurred in basal cells while suprabasal cells became oval-shaped in the medial region. Cells remained columnar in the lateral region (Figures 2B,B’). At E13.5, basal cells in both the lateral and medial regions became columnar with basal constriction, while hinge cells were with apical constriction (Figures 2C,C’).

To understand whether cell shape changes result in epithelial folding, we measured the apical and basal widths of basal cells (apical/basal ratio = apical width/basal width indicated aw-aw’/b-b’) (Figure 2D). At E12.5, all basal cells were columnar with apical/basal ratio ≈1 (Figures 2A,A’,D’). At E13.0, the apical/basal ratio of mediobasal cells increased (>1), due to the basal width reduction (Figures 2B,B’,D’). At E13.5, basal cells were categorized by the apical/basal ratio into two groups: cells in the hinge region with apical constriction (<1) and cells in the medial and lateral regions with basal constriction (>1) (Figures 2C,C’,D’). To understand whether cell elongation had occurred, the width/length ratio (b-b’/a-a’) was calculated (Figures 2E,E’). At E12.5, suprabasal cells were mostly elongated (width/length ratio ≈0.5) (Figures 2A,A’,E’). However, suprabasal cells in the medial region became oval-shaped at E13.0 (≈1) while those in the lateral region remained elongated (≈0.5) (Figures 2B,B’,E’). Overlaying the novel placode-to-invagination mechanism with the suprabasal intercalation model requires the existence of suprabasal elongated cells positioned in the direction parallel to the basal lamina to generate force for invagination (Panousopoulou and Green, 2016). To investigate whether suprabasal intercalation occurs in the placode to dome-shape transition, the orientations of suprabasal cells at E12.5 and E13.0 were measured (Figures 2F,F’). The angles of suprabasal cells (long axis) to the plane of the flanking epithelia were all in the vertical-to-oblique range (i.e., 45°–135°) at E12.5 (80°–100°) and E13.0 (70°–110°). Each suprabasal cell was not parallel to the plane of the flanking epithelium (Figure 2F’). Thus, suprabasal intercalation did not occur; the force of invagination could not be generated.

### Actomyosin-Dependent Cellular Morphology

**FIGURE 3:**
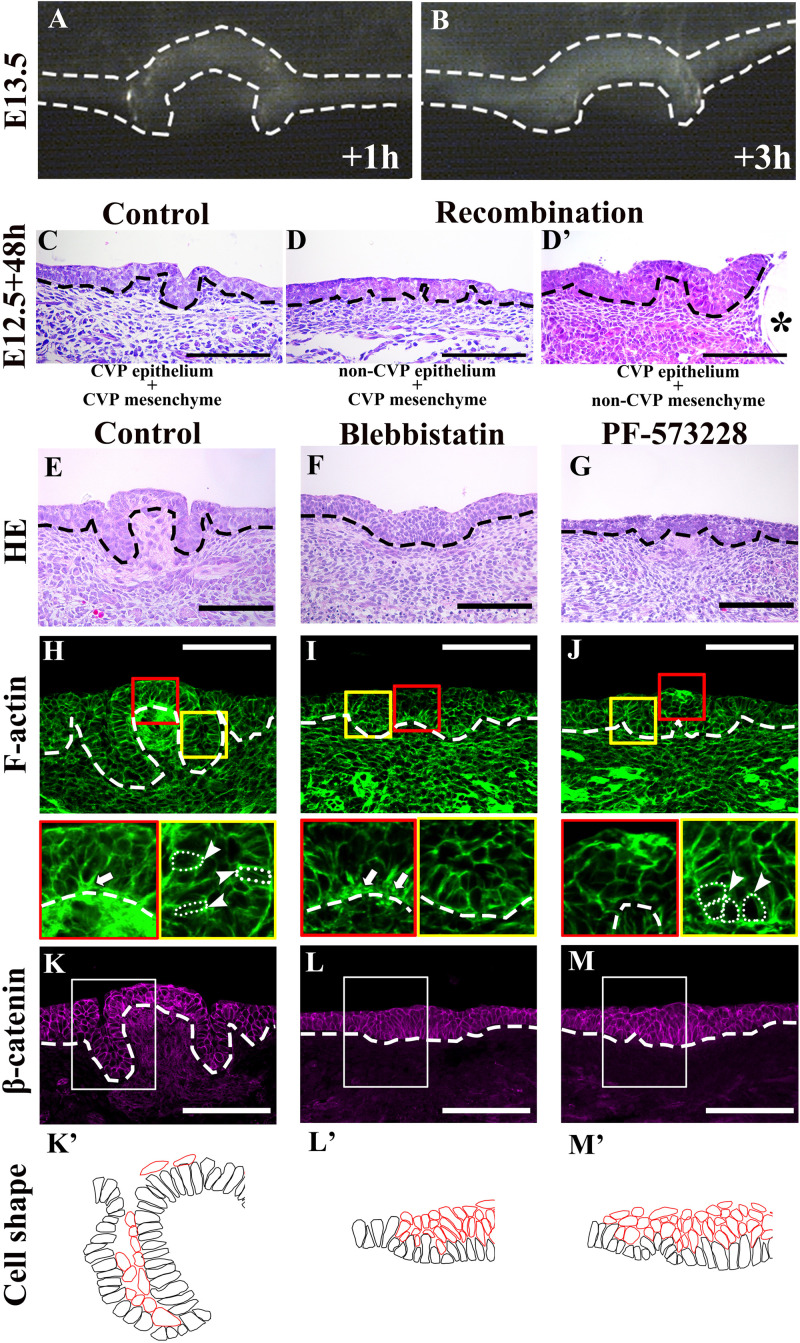
**(A,B)** Original dome-shaped and trenches of the detached CVP epithelium retain their own structure after 1 and 3 h of in vitro culture at E13.5. **(C)** After recombination at E12.5 with the CVP epithelium and CVP mesenchyme, dome-shaped and trench structures are observed after 48 h culture. **(D)** Epithelial folding is not observed in the recombination of non-CVP epithelium and CVP mesenchyme group. **(D’)** Similar epithelial folding is found in the recombination of CVP epithelium and non-CVP mesenchyme with FGF10-soaked bead. The asterisk indicates the FGF10-soaked beads for the maintenance of Lgr5-positive epithelial stem cells in CVP (Zhang et al., 2018). **(E)** E12.5 + 48 h cultured control group CVP morphology is similar to E13.5 in vivo. **(F)** The Blebbistatin-treated group shows disrupted invagination and evagination, and the CVP epithelium remains stratified. **(G)** The PF-573228-treated group shows shallow trenches and CVP placode in the dome-shape region are observed. **(H)** Basal F-actin accumulation at the dome-shaped region and apical accumulation of F-actin at the trench region are observed in control (arrow and arrowhead; compared to Figure 1R). **(I)** In the Blebbistatin-treated group, both basal accumulation (arrow) and apical F-actin accumulation are reduced. **(J)** In the PF-573228-treated group, apical accumulation of F-actin exists in the trench region (arrowhead), but basal F-actin accumulation is not observed. **(K,K’)** Most basal cells are elongated in the control group, similar to those at E13.5 in vivo. **(L,L’)** Mediobasal cells show disrupted basal constriction and cells in the trench region remain oval-shape in the Blebbistatin-treated group. **(M,M’)** In the PF-573228-treated group, basal cell shape in the trench region are similar to the control. Basal cells in the dome-shape region are columnar with apical constriction instead of basally constriction. Scale bar = 100 μm. Arrowheads indicate the apical accumulation of F-actin. Arrows indicate basal accumulation of F-actin. Thick dotted lines mark the border between developing tongue epithelium and mesenchyme. Asterisks indicate FGF10 soaked beads. Thin white dotted circles indicate individual cell shape which goes through apical/basal constriction.

In order to examine whether epithelial folding is mechanically independent of the underlying mesenchyme, the CVP epithelium was separated from the mesenchyme at E13.5. Detached CVP epithelium maintained its original structure after in vitro culture for 1 and 3 h; this revealed that the mesenchyme was not mechanically required (Figures 3A,B). To determine whether epithelial folding depends on epithelial cells, reverse recombination was performed at E12.5 for 48 h. As we have shown previously, the CVP epithelium and non-CVP mesenchyme recombination group showed an absence of CVP structure. For rescue experiment, FGF10-soaked beads were implanted in the recombinant CVP epithelium and non-CVP mesenchyme for the maintenance of Lgr5-positive epithelial cells (Zhang et al., 2018). Epithelial folding was observed when CVP epithelium and the mesenchyme were recombined (Figure 3C), but absent when non-CVP epithelium was recombined with CVP mesenchyme (Figure 3D). To further clarify the role of CVP mesenchyme in epithelial folding, CVP epithelium, and non-CVP mesenchyme were recombined with FGF10-soaked beads. Epithelial folding morphology was similar to that seen in CVP epithelium and mesenchyme recombination, indicating that epithelial folding might be dependent on epithelial cell shape changes (Figure 3D’). Blebbistatin binds to the ATPase intermediate with ADP and phosphate bound to the active site and slows down the subsequent phosphorylation of myosin light chain II (Kovács et al., 2004). PF-573228 works as an FAK phosphorylation inhibitor on Tyr^397^ (Slack-Davis et al., 2007). Blebbistatin and PF-573228 were introduced to reveal the roles of actomyosin contraction and FAK in CVP morphogenesis. In tongue from E12.5 cultured for 48 h, the control group with vehicle (DMSO) developed into a dome-shape similar to that seen at stage E13.5 in vivo (Figure 3E). In the Blebbistatin-treated group, the epithelium remained stratified while both invagination and evagination were disrupted (Figure 3F). In the PF-573228-treated group, even invagination was reduced; shallow trenches were observed. However, evagination was arrested and CVP placodes were observed in the dome-shaped region (Figure 3G). F-actin accumulated at the basal site of mediobasal cells (arrow) and the apical site of laterobasal cells (arrowhead) in control (Figure 3H). F-actin accumulation in basal sites of medial cells was reduced after Blebbistatin treatment (Figure 3I, arrow). In the PF-573228-treated group, F-actin accumulation remained at the apical site in the trench region (arrowhead), but the basal accumulation in mediobasal cells was completely diminished (Figure 3J). The differences in F-actin intensity between apical and basal sites of basal cells were evident in the control group, but diminished after treatment with Blebbistatin and PF-573228 (Table 1). β-catenin staining showed that cells in both dome-shaped and trench regions were elongated in the control group (Figures 3K,K’). In the Blebbistatin-treated group, basal constriction was reduced in the dome-shaped region. In the trench region, cells remained oval-shaped instead of elongated (Figures 3L,L’). The shapes of basal cells in trenches were similar to those of the control after PF-573228 treatment, but basal cells in the medial region remained apical-constricted or columnar (Figures 3M,M’). These results indicated that actomyosin contraction is necessary for apical/basal constriction of epithelial cells. Thus, subsequent epithelial folding was disrupted after Blebbistatin treatment. Moreover, basal constriction in the medial region was highly dependent on FAK.

### Shh Regulates Trench and Stromal Core Formation

**FIGURE 4:**
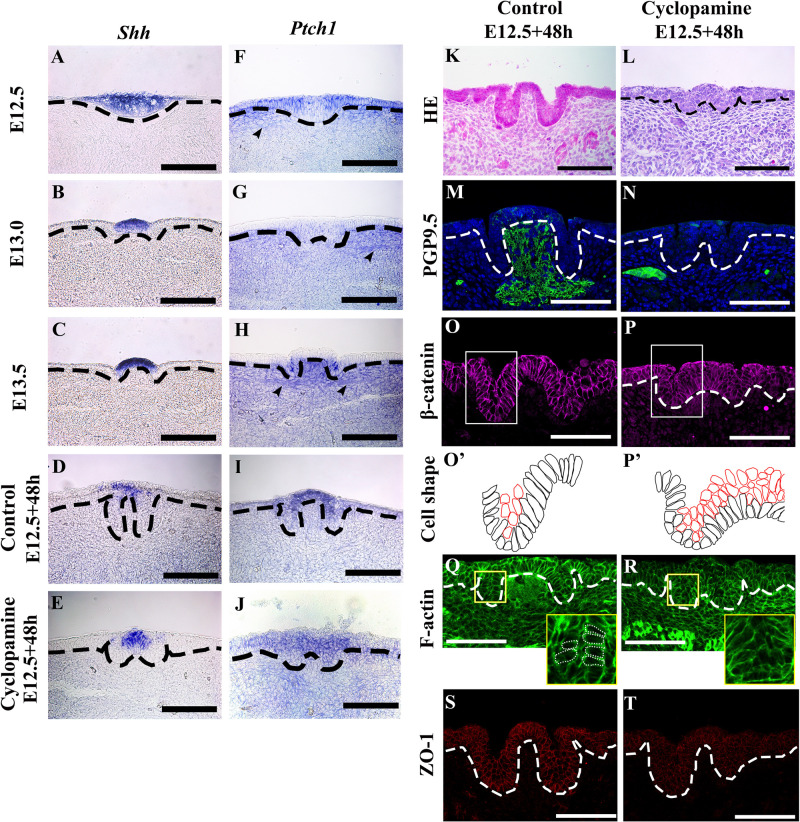
**(A)** Sonic hedgehog (Shh) expression is observed in the whole CVP placode at E12.5. **(B,C)** Shh expression is detected in the apical region of the dome-shape epithelium at E13.0 and E13.5. **(D,E)** In the E12.5 + 48 h cultured control group and Cyclopamine-treated group, expression patterns of Shh are similar to E13.5 CVP in vivo. **(F)** At E12.5, Ptch1 is expressed in the lateral region of the CVP placode, non-CVP epithelium, and underlying mesenchyme. **(G)** The expression of Ptch1 is weakly detected in CVP at E13.0 compared to at E12.5. **(H)** Epithelial cells in the trench region and stromal core show a strong expression of Ptch1 at E13.5. **(F–H)** The arrowheads indicate Ptch1-positive neuronal components from E12.5 to E13.5. **(I,J)** In the E12.5 + 48 h cultured control and Cyclopamine-treated groups, Ptch1 is strongly expressed in the apical region of the dome-shape compared to the non-CVP epithelium. **(K,L)** Compared to the control group, the Cyclopamine-treated group has abnormal trenches and a stratified CVP epithelium with disrupted stromal core. **(M,N)** The stromal core in the Cyclopamine-treated group shows the absence of PGP9.5-positive neuronal components while strong localization is observed in control group. **(O,P,O’,P’)** β-catenin staining shows that mediobasal cells in the dome-shaped region show basal constriction in both control and Cyclopamine-treated group. Cells in the trench region are not elongated but remain oval-shape in the Cyclopamine-treated group. Suprabasal cells remain in the Cyclopamine-treated group. **(Q,R)** Phalloidin staining shows that the apical accumulation of F-actin in trench region is reduced after the Cyclopamine treatment while basal accumulation of F-actin in dome-shape region is similar as control. **(S,T)** Tight junction protein 1 (ZO-1) is strongly located in epithelial cells in the control group, but reduced in the Cyclopamine-treated group. Scale bar = 100 μm. Arrowheads indicate neuronal components. Thick dotted lines mark the border between developing tongue epithelium and mesenchyme. Thin dotted circles indicate individual cell shape which goes through apical/basal constriction.

To investigate the roles of Shh and Ptch1 on epithelial folding and stromal core formation, we examined Shh and Ptch1 expression patterns in developing CVP. Shh was expressed in the CVP placode at E12.5 and the apical region of the dome-shaped epithelium at E13.0 and E13.5 (Figures 4A–C). Ptch1 was expressed in the lateral region of the placode in non-CVP epithelium and the underlying mesenchyme at E12.5 (Figure 4F). At E13.0, Ptch1 was faintly expressed in the CVP epithelium (Figure 4G). Strong Ptch1 expression was found within the trench region epithelium and the stromal core at E13.5 (Figure 4H). Similar to the localization of ETB expression, neuronal components were also marked by Ptch1 from E12.5 to E13.5 (Figures 4F–H). To understand Shh pathway function in CVP morphogenesis, Cyclopamine was introduced into an in vitro culture for 48 h. Similar to CVP at E13.5, Shh was expressed in the apical region of the dome-shaped epithelium in both control and Cyclopamine-treated groups (Figures 4D,E). Both in control and Cyclopamine-treated groups, Ptch1 expression was detected within the apical region of the CVP epithelium and detected more weakly in the trench region (Figures 4I,J). Moreover, Cyclopamine-treated groups showed defects in trench formation within the stratified epithelium and exhibited disrupted stromal cores (Figure 4L) compared to the control (Figure 4K). PGP9.5-positive neuronal components were absent in the stromal core after Cyclopamine treatment, which compared starkly to strong PGP9.5 localization in the control group (Figures 4M,N). Mediobasal cells showed basal constriction in the dome-shaped region of both the control and Cyclopamine-treated groups. Cells in the trench region were oval-shaped, not columnar, while trench formation was disrupted after Cyclopamine treatment (Figures 4O,P,O’,P’). According to the Blebbistatin-inhibited actomyosin contraction in the trench region, we hypothesized that trench formation is regulated via cytoskeletal alteration through Shh and its downstream signaling. F-actin accumulation was detected at the basal site of mediobasal cells in both control and Cyclopamine-treated groups (Figures 4Q,R). However, apical accumulation of F-actin in the trench region was reduced after Cyclopamine treatment (Yellow panels in Figures 4Q,R), which corresponds with disrupted cell shape changes in trench epithelial cells (Figures 4P,P’). The differences in F-actin intensity between apical and basal sites of trench regions were disrupted in the Cyclopamine-treated group (Table 1). ZO-1 strongly localized within the epithelial cells in the control group (Figure 4S). Conversely, ZO-1 was reduced in the Cyclopamine-treated group (Figure 4T). These results indicated that Shh signaling regulated trench formation through actomyosin-dependent apical constriction. Finally, stromal core formation was affected by Shh the modulating survival or differentiation of neurons in CVP ganglion.

This study demonstrated that the transition of CVP from placode to dome-shape required actomyosin-dependent epithelial folding invagination and evagination. Moreover, the occurrence of evagination in the dome-shaped formations required FAK for basal constriction Trench and stromal core formations were interrupted as a result of inhibited Shh pathway. In the process of invagination during the formation of trenches, a correlation was demonstrated between the presence of Shh and apical constriction of trench epithelial cells. Furthermore, the differentiation or survival of neurons in CVP ganglion might be impacted by epithelial Shh in the formation of stromal core.

## Discussion

This study demonstrated that CVP placode to dome-shape transition required actomyosin-dependent epithelial folding including invagination and evagination. Moreover, evagination in dome-shape formation required FAK for basal constriction. Trench and stromal core formation were interrupted after inhibition of Shh pathway by Cyclopamine. Furthermore, epithelial *Shh* also showed impact in stromal core formation of developing CVP.

Previously, multiple epithelial folding mechanisms have been reported, including actions on both monolayered and multilayered epithelium (Sai and Ladher, 2008; Sawyer et al., 2010; Martin and Goldstein, 2014; Li et al., 2016; Panousopoulou and Green, 2016; Pearl et al., 2017). Multilayered CVP placodes lack parallel-oriented suprabasal cells with specific E-cadherin localization. The morphology indicated that previously considered epithelial folding mechanisms were not responsible for the transitions in developing CVP (Panousopoulou and Green, 2016; Pearl et al., 2017). Therefore, we analyzed cell shape changes to investigate the underlying mechanisms of epithelial folding (Pearl et al., 2017). Cell shape analysis indicated that basal constriction occurred in the evagination region and apical constriction occurred in the invagination region (Figure 2D’). F-actin and pMLC staining in developing CVP (Figures 1S–X) corresponded with the co-accumulation of F-actin and pMLC observed in basal and apical constriction sites. This indicated that cell shape changes might depend on actomyosin. To further confirm whether cell shape changes are actomyosin-dependent, actomyosin was inhibited by Blebbistatin and epithelial folding was sufficiently disrupted (Figures 3F,I,L,L’).

Since both evagination and invagination were instigated by different types of cell shape changes (basal and apical constriction) and were actomyosin-dependent, we investigated whether the previously reported actomyosin-dependent mechanisms applied in the case of dome-shape formation. Outward folding activated by actomyosin-dependent basal constriction has been found FAK-dependent in the formation of the midbrain-hindbrain boundary (Gutzman et al., 2008, 2018). Recently, it has also been demonstrated that this mechanism is conserved in the bud-to-cap transition in tooth development, which suggested that evagination in developing CVP might be controlled by similar mechanisms (Yamada et al., 2019). We showed that inhibition of FAK was sufficient to arrest basal constriction and subsequent evagination and indicated that FAK-dependent basal constriction is involved in the formation of dome-shaped CVP (Figures 3G,J,M,M’). This result suggested that the factors involved in the midbrain-hindbrain boundary and tooth bud-to-cap transition might be active in developing CVP (Gutzman et al., 2008; Pearl et al., 2017; Yamada et al., 2019).

We further investigated factors with the potential to regulate apical constriction. Previously research indicates that Shh could play a large regulatory role in CVP morphogenesis. Shh was continuously expressed from the placode stage through the dome-shaped stage in developing CVP (Li et al., 2016). *Shh* was continuously expressed from the placode stage through the dome-shaped stage in developing CVP (Hall et al., 1999, 2003; Liu et al., 2004; Lee et al., 2006; Iwatsuki et al., 2007). Inhibition experiments of Shh have been conducted previously, but morphological defects within CVP has not been reported (Hall et al., 1999, 2003; Liu et al., 2004; Lee et al., 2006; Iwatsuki et al., 2007; Kim et al., 2009). To pinpoint the impact of Shh, we conducted the experiments herein. Based on our results, inhibition of Shh pathway disrupted both trench and stromal core formation in developing CVP (Figures 4L,N,P). The abnormal trenches (Figure 4L) after inhibition of Shh pathway were similar to the Cyclopamine-treated tooth placodes and hair placodes with cell intercalation defects (Ahtiainen et al., 2014; Li et al., 2016). Disrupted apical accumulation of F-actin in the Cyclopamine-treated CVP indicated that the morphological defect was caused by the interrupted cytoskeletal alteration (Figure 4R). Since Ptch1 was expressed at the lateral region of the placode at E13.0 and trench epithelium at E13.5 (Figures 4G,H), Shh expressed at the apical region of CVP epithelium possesses a potential regulatory role in cytoskeletal alteration in trench epithelial cells and subsequent trench formation. Support for this hypothesis comes concurrently from the impaired trench formation by the reduction of Shh expression in *Pax9*^–/–^ mice (Kist et al., 2014). Moreover, ZO-1 has been reported to bind directly to F-actin and determine epithelial polarity (Itoh et al., 1997; Fanning et al., 1998; Odenwald et al., 2017). ZO-1 was reduced in CVP trench epithelial cells after Cyclopamine treatment during CVP morphogenesis.

A disrupted stromal core lacking neuronal components was observed after Cyclopamine treatment (Figure 4N). Epithelial Shh has been reported as a regulator of neuron formation through its modulation of cell migration in the development of the enteric nervous system (Baynash et al., 1994; Hosoda et al., 1994; Nagy et al., 2016). We observed that the neuronal components in the stromal core were marked by ETB, an enteric nervous system marker, as well as PGP9.5 (Figures 1D–F). In addition to this finding, Neurogenin2 was expressed in the underlying mesenchyme (Supplementary Figures S1J–K), which marks neuroblasts (Fode et al., 1998; Okubo and Takada, 2015; Fan et al., 2019). *Shh* supposed to involve in stromal core formation via the regulation of neuroblasts- and NCCs-derived cell.

Previous studies reported that cell migration regulated by Shh occurs through a Gli-Smoothened pathway-independent mechanism (Testaz et al., 2001; Nagy et al., 2016). Though neuroblasts and NCCs-derived cells in the stromal core were Ptch1 positive (Figures 4F–H), Gli1, Gli2, and Gli3 expression were not detected in the stromal core of CVP from E12.5 to E13.5 (data not shown). The absence of Gli1, Gli2 and Gli3 indicated that the Gli-Smoothened pathway might not be involved in the stromal core formation from E12.5 to E13.5.

**FIGURE 5:**
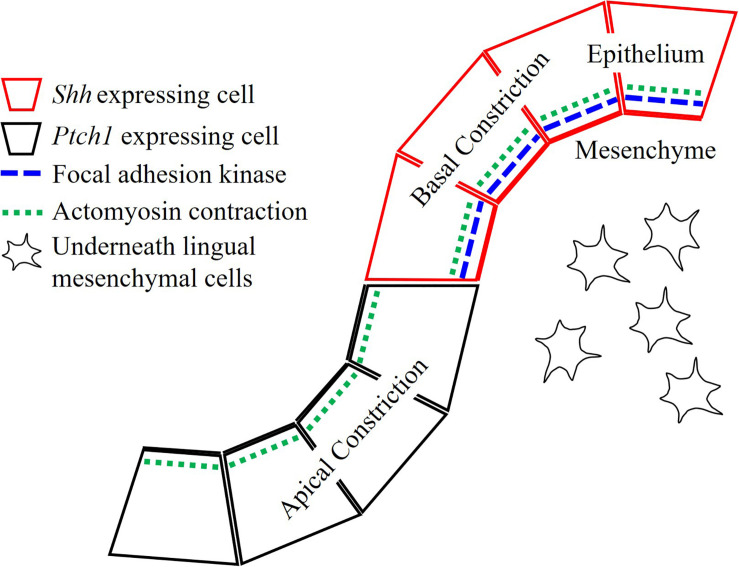
Actomyosin contraction is essential for both dome-shape and trench formation. Actomyosin-dependent basal constriction in the dome-shape region requires focal adhesion kinase. Epithelial Shh regulates cytoskeletal alteration in the trench formation and differentiation or survival of neurons in stromal core of CVP.

In summary, the epithelial folding seen in the transition from placode to dome-shape was actomyosin-dependent (Figure 5). FAK-dependent basal constriction in the medial region led to evagination, which formed the dome-shape. Shh-guided apical constriction in the lateral region led trenches to form via invagination. Moreover, stromal core formation is also under regulation of Shh pathway.

## Data Availability Statement

The raw data supporting the conclusions of this article will be made available by the authors, without undue reservation.

## Ethics Statement

The animal study was reviewed and approved by the Intramural Animal Use and Care Committee of the College of Dentistry, Yonsei University.

## Author Contributions

SZ, J-ML, and H-SJ designed and performed the experiments and analysis. AA performed the analysis of data. All authors contributed to manuscript revision, read and approved the submitted version.

## Conflict of Interest

The authors declare that the research was conducted in the absence of any commercial or financial relationships that could be construed as a potential conflict of interest.

## Supplementary Material

FIGURE S1 **(A)** The neuronal marker PGP9.5 and enteric nervous system precursor marker endothelin receptor B (ETB) are co-localized in the underlying mesenchyme of CVP at E13.0. **(B)** NCAM is located in the stromal core of CVP at E13.5. **(C)** Fibronectin is weakly detected in ETB located underlying mesenchyme of CVP at E13.0. **(D)** At E12.5, Ki67-positive proliferating cells are absent in the CVP placode. **(E)** Ki67-positive cells retain in flanking non-CVP epithelium but reduce in the CVP mesenchyme at E13.0. At E13.5, proliferative cells appear in the trench region but not in the dome-shaped region epithelium. **(F)** Ki67-positive cells are located in the stromal core of CVP at E13.5. **(G–I)** There is no apoptotic cell in developing CVP epithelium and mesenchyme from E12.5 to E13.5 (Lee et al.). There is no apoptotic cell in developing CVP epithelium and mesenchyme from E12.5 to E13.5 (Lee et al.). **(J,K)** Neurogenin2 is expressed in neuronal components located in the underlying mesenchyme of developing CVP at E12.5 and E13.0. Scale bar = 100 μm. White/black dotted lines mark the border between developing tongue epithelium and mesenchyme. Arrowheads indicates neuronal components.

## References

[B1] Ahtiainen L. Lefebvre S. Lindfors P. H. Renvoisé E. Shirokova V. Vartiainen M. K. (2014). Directional cell migration, but not proliferation, drives hair placode morphogenesis. *Dev. Cell* 28 588–602. 10.1016/j.devcel.2014.02.003 24636260

[B2] Barlow L. A. Klein O. D. (2015). Developing and regenerating a sense of taste. *Curr. Top. Dev. Biol.* 111 401–419. 10.1016/bs.ctdb.2014.11.012 25662267 PMC4435577

[B3] Baynash A. G. Hosoda K. Giaid A. Richardson J. A. Emoto N. Hammer R. E. (1994). Interaction of endothelin-3 with endothelin-B receptor is essential for development of epidermal melanocytes and enteric neurons. *Cell* 79 1277–1285. 10.1016/0092-8674(94)90018-3 8001160

[B4] Beites C. L. Hollenbeck P. L. Kim J. Lovell-Badge R. Lander A. D. Calof A. L. (2009). Follistatin modulates a BMP autoregulatory loop to control the size and patterning of sensory domains in the developing tongue. *Development* 136 2187–2197. 10.1242/dev.030544 19474151 PMC2729339

[B5] Chandrashekar J. Hoon M. A. Ryba N. J. Zuker C. S. (2006). The receptors and cells for mammalian taste. *Nature* 444 288–294. 10.1038/nature05401 17108952

[B6] Fan D. Chettouh Z. Consalez G. G. Brunet J. F. (2019). Taste bud formation depends on taste nerves. *eLife* 8:e49226. 10.7554/eLife.49226 31570121 PMC6785267

[B7] Fanning A. S. Jameson B. J. Jesaitis L. A. Anderson J. M. (1998). The tight junction protein ZO-1 establishes a link between the transmembrane protein occludin and the actin cytoskeleton. *J. Biol. Chem.* 273 29745–29753. 10.1074/jbc.273.45.29745 9792688

[B8] Fode C. Gradwohl G. Morin X. Dierich A. LeMeur M. Goridis C. (1998). The bHLH protein NEUROGENIN 2 is a determination factor for epibranchial placode–derived sensory neurons. *Neuron* 20 483–494. 10.1016/s0896-6273(00)80989-7 9539123

[B9] Guth L. (1957). The effects of glossopharyngeal nerve transection on the circumvallate papilla of the rat. *Anat. Rec.* 128 715–731. 10.1002/ar.1091280406 13478886

[B10] Gutzman J. H. Graeden E. Brachmann I. Yamazoe S. Chen J. K. Sive H. (2018). Basal constriction during midbrain–hindbrain boundary morphogenesis is mediated by Wnt5b and focal adhesion kinase. *Biol. Open* 7:bio034520. 10.1242/bio.034520 30305282 PMC6262868

[B11] Gutzman J. H. Graeden E. G. Lowery L. A. Holley H. S. Sive H. (2008). Formation of the zebrafish midbrain–hindbrain boundary constriction requires laminin-dependent basal constriction. *Mech. Dev.* 125 974–983. 10.1016/j.mod.2008.07.004 18682291 PMC2780020

[B12] Hall J. M. Bell M. L. Finger T. E. (2003). Disruption of sonic hedgehog signaling alters growth and patterning of lingual taste papillae. *Dev. Biol.* 255 263–277. 10.1016/s0012-1606(02)00048-9 12648489

[B13] Hall J. M. Hooper J. E. Finger T. E. (1999). Expression of sonic hedgehog, patched, and Gli1 in developing taste papillae of the mouse. *J. Comp. Neurol.* 406 143–155. 10.1002/(sici)1096-9861(19990405)406:2<143::aid-cne1>3.0.co;2-x 10096602

[B14] Harlow D. E. Barlow L. A. (2007). Embryonic origin of gustatory cranial sensory neurons. *Dev. Biol.* 310 317–328. 10.1016/j.ydbio.2007.07.042 17826760 PMC2078608

[B15] Hosoda K. Hammer R. E. Richardson J. A. Baynash A. G. Cheung J. C. Giaid A. (1994). Targeted and natural (piebald-lethal) mutations of endothelin-B receptor gene produce megacolon associated with spotted coat color in mice. *Cell* 79 1267–1276. 10.1016/0092-8674(94)90017-5 8001159

[B16] Itoh M. Nagafuchi A. Moroi S. Tsukita S. (1997). Involvement of ZO-1 in cadherin-based cell adhesion through its direct binding to α catenin and actin filaments. *J. Cell Biol.* 138 181–192. 10.1083/jcb.138.1.181 9214391 PMC2139940

[B17] Iwatsuki K. Liu H.-X. Grónder A. Singer M. A. Lane T. F. Grosschedl R. (2007). Wnt signaling interacts with Shh to regulate taste papilla development. *Proc. Natl. Acad. Sci. U.S.A.* 104 2253–2258. 10.1073/pnas.0607399104 17284610 PMC1794217

[B18] Jaskoll T. Leo T. Witcher D. Ormestad M. Astorga J. Bringas P. Jr. (2004). Sonic hedgehog signaling plays an essential role during embryonic salivary gland epithelial branching morphogenesis. *Dev. Dyn.* 229 722–732. 10.1002/dvdy.10472 15042696

[B19] Jitpukdeebodintra S. Chai Y. Snead M. L. (2003). Developmental patterning of the circumvallate papilla. *Int. J. Dev. Biol.* 46 755–763. 12216988

[B20] Kim J. Y. Lee M. J. Cho K. W. Lee J. M. Kim Y. J. Kim J. Y. (2009). Shh and ROCK1 modulate the dynamic epithelial morphogenesis in circumvallate papilla development. *Dev. Biol.* 325 273–280. 10.1016/j.ydbio.2008.10.034 19014928

[B21] Kist R. Watson M. Crosier M. Robinson M. Fuchs J. Reichelt J. (2014). The formation of endoderm-derived taste sensory organs requires a Pax9-dependent expansion of embryonic taste bud progenitor cells. *PLoS Genet.* 10:e1004709. 10.1371/journal.pgen.1004709 25299669 PMC4191947

[B22] Kovács M. Tóth J. Hetényi C. Málnási-Csizmadia A. Sellers J. R. (2004). Mechanism of blebbistatin inhibition of myosin II. *J. Biol. Chem.* 279 35557–35563. 10.1074/jbc.m405319200 15205456

[B23] Lee M.-J. Kim J.-Y. Lee S.-I. Sasaki H. Lunny D. P. Lane E. B. (2006). Association of Shh and Ptc with keratin localization in the initiation of the formation of circumvallate papilla and von Ebner’s gland. *Cell Tissue Res.* 325:253. 10.1007/s00441-006-0160-1 16552524

[B24] Li J. Chatzeli L. Panousopoulou E. Tucker A. S. Green J. B. (2016). Epithelial stratification and placode invagination are separable functions in early morphogenesis of the molar tooth. *Development* 143 670–681. 10.1242/dev.130187 26755699 PMC4760321

[B25] Liu F. Thirumangalathu S. Gallant N. M. Yang S. H. Stoick-Cooper C. L. Reddy S. T. (2007). Wnt-β-catenin signaling initiates taste papilla development. *Nat. Genet.* 39:106. 10.1038/ng1932 17128274

[B26] Liu H. X. Komatsu Y. Mishina Y. Mistretta C. M. (2012). Neural crest contribution to lingual mesenchyme, epithelium and developing taste papillae and taste buds. *Dev. Biol.* 368 294–303. 10.1016/j.ydbio.2012.05.028 22659543 PMC3402632

[B27] Liu H.-X. MacCallum D. K. Edwards C. Gaffield W. Mistretta C. M. (2004). Sonic hedgehog exerts distinct, stage-specific effects on tongue and taste papilla development. *Dev. Biol.* 276 280–300. 10.1016/j.ydbio.2004.07.042 15581865

[B28] Martin A. C. Goldstein B. (2014). Apical constriction: themes and variations on a cellular mechanism driving morphogenesis. *Development* 141 1987–1998. 10.1242/dev.102228 24803648 PMC4011084

[B29] Meng W. Takeichi M. (2009). Adherens junction: molecular architecture and regulation. *Cold Spring Harb. Perspect. Biol.* 1:a002899. 10.1101/cshperspect.a002899 20457565 PMC2882120

[B30] Mistretta C. M. Liu H.-X. Gaffield W. MacCallum D. K. (2003). Cyclopamine and jervine in embryonic rat tongue cultures demonstrate a role for Shh signaling in taste papilla development and patterning: fungiform papillae double in number and form in novel locations in dorsal lingual epithelium. *Dev. Biol.* 254 1–18. 10.1016/s0012-1606(02)00014-3 12606278

[B31] Nagy N. Barad C. Graham H. K. Hotta R. Cheng L. S. Fejszak N. (2016). Sonic hedgehog controls enteric nervous system development by patterning the extracellular matrix. *Development* 143 264–275. 10.1242/dev.128132 26674309 PMC4725345

[B32] Nosrat I. V. Margolskee R. F. Nosrat C. A. (2012). Targeted taste cell-specific overexpression of brain-derived neurotrophic factor in adult taste buds elevates phosphorylated TrkB protein levels in taste cells, increases taste bud size, and promotes gustatory innervation. *J. Biol. Chem.* 287 16791–16800. 10.1074/jbc.m111.328476 22442142 PMC3351349

[B33] Odenwald M. A. Choi W. Buckley A. Shashikanth N. Joseph N. E. Wang Y. (2017). ZO-1 interactions with F-actin and occludin direct epithelial polarization and single lumen specification in 3D culture. *J. Cell Sci.* 130 243–259. 10.1242/jcs.188185 27802160 PMC5394778

[B34] Okubo T. Takada S. (2015). Pharyngeal arch deficiencies affect taste bud development in the circumvallate papilla with aberrant glossopharyngeal nerve formation. *Dev. Dyn.* 244 874–887. 10.1002/dvdy.24289 25997579

[B35] Panousopoulou E. Green J. B. (2016). Invagination of ectodermal placodes is driven by cell intercalation-mediated contraction of the suprabasal tissue canopy. *PLoS Biol.* 14:e1002405. 10.1371/journal.pbio.1002405 26960155 PMC4784948

[B36] Pearl E. J. Li J. Green J. B. (2017). Cellular systems for epithelial invagination. *Philos. Trans. R. Soc. Lond. B Biol. Sci.* 372:20150526. 10.1098/rstb.2015.0526 28348256 PMC5379028

[B37] Petersen C. I. Jheon A. H. Mostowfi P. Charles C. Ching S. Thirumangalathu S. (2011). FGF signaling regulates the number of posterior taste papillae by controlling progenitor field size. *PLoS Genet.* 7:e1002098. 10.1371/journal.pgen.1002098 21655085 PMC3107195

[B38] Sai X. Ladher R. K. (2008). FGF signaling regulates cytoskeletal remodeling during epithelial morphogenesis. *Curr. Biol.* 18 976–981. 10.1016/j.cub.2008.05.049 18583133

[B39] Sawyer J. M. Harrell J. R. Shemer G. Sullivan-Brown J. Roh-Johnson M. Goldstein B. (2010). Apical constriction: a cell shape change that can drive morphogenesis. *Dev. Biol.* 341 5–19. 10.1016/j.ydbio.2009.09.009 19751720 PMC2875788

[B40] Schindelin J. Arganda-Carreras I. Frise E. Kaynig V. Longair M. Pietzsch T. (2012). Fiji: an open-source platform for biological-image analysis. *Nat. Methods* 9:676. 10.1038/nmeth.2019 22743772 PMC3855844

[B41] Slack-Davis J. K. Martin K. H. Tilghman R. W. Iwanicki M. Ung E. J. Autry C. (2007). Cellular characterization of a novel focal adhesion kinase inhibitor. *J. Biol. Chem.* 282 14845–14852. 17395594 10.1074/jbc.M606695200

[B42] Suzuki Y. Ikeda K. Kawakami K. (2011). Development of gustatory papillae in the absence of Six1 and Six4. *J. Anat.* 219 710–721. 10.1111/j.1469-7580.2011.01435.x 21978088 PMC3237879

[B43] Testaz S. Jarov A. Williams K. P. Ling L. E. Koteliansky V. E. Fournier-Thibault C. (2001). Sonic hedgehog restricts adhesion and migration of neural crest cells independently of the Patched-Smoothened-Gli signaling pathway. *Proc. Natl. Acad. Sci. U.S.A.* 98 12521–12526. 10.1073/pnas.221108698 11592978 PMC60086

[B44] Yamada S. Lav R. Li J. Tucker A. S. Green J. B. A. (2019). Molar Bud-to-Cap Transition Is Proliferation Independent. *J. Dent. Res.* 98 1253–1261. 10.1177/0022034519869307 31393749 PMC6761786

[B45] Zhang S. Choi H. S. Jung H.-S. Lee J.-M. (2018). FGF10 is required for circumvallate papilla morphogenesis by maintaining Lgr5 activity. *Front. Physiol.* 9:1192. 10.3389/fphys.2018.01192 30233392 PMC6127645

[B46] Zhou Y. Liu H.-X. Mistretta C. M. (2006). Bone morphogenetic proteins and noggin: inhibiting and inducing fungiform taste papilla development. *Dev. Biol.* 297 198–213. 10.1016/j.ydbio.2006.05.022 16828469

